# *OTUD7B* upregulation predicts a poor response to paclitaxel in patients with triple-negative breast cancer

**DOI:** 10.18632/oncotarget.23074

**Published:** 2017-12-09

**Authors:** Hui-Wen Chiu, Hui-Yu Lin, Ing-Jy Tseng, Yuan-Feng Lin

**Affiliations:** ^1^ Graduate Institute of Clinical Medicine, College of Medicine, Taipei Medical University, Taipei, Taiwan; ^2^ Division of Nephrology, Department of Internal Medicine, Shuang Ho Hospital, Taipei Medical University, Taiwan; ^3^ Department of Breast Surgery and General Surgery, Division of Surgery, Cardinal Tien hospital, Xindian District, New Taipei City, Taiwan; ^4^ Gerontology Health Management, College of Nursing, Taipei Medical University, Taipei, Taiwan

**Keywords:** OTUD7B, paclitaxel, chemotherapy, in silico analysis, triple-negative breast cancer

## Abstract

Paclitaxel is a first-line chemotherapeutic for patients with breast cancer, particularly triple-negative breast cancer (TNBC). Molecular markers for predicting pathologic responses to paclitaxel treatment is thus urgently needed since paclitaxel resistance is still a clinical issue in treating TNBCs. We investigated the transcriptional profiling of consensus genes in HCC38 (paclitaxel-sensitive) and MDA-MB436 (paclitaxel-resistant) TNBC cells post-treatment with paclitaxel. We found that *OTUD7B* was downregulated in HCC38 but upregulated in MDA-MB436 cells after paclitaxel treatment at cytotoxic concentrations. Moreover, our data showed that *OTUD7B* expression causally correlated with IC_50_ of paclitaxel in a panel of TNBC cell lines. Moreover, we found that *OTUD7B* upregulation was significantly detected in primary breast cancer tissues compared to normal breast tissues but inversely correlated with tumor growth in TNBC cells. Besides, the increased levels of OTUD7B transcript appeared to causally associate with invasive potentials in TNBC cells. In assessments of recurrence/metastasis-free survival probability, high-levels of *OTUD7B* transcripts strongly predicted a poor prognosis and unfavorable response to paclitaxel-based chemotherapy in patients with TNBCs. *In silico* analysis suggested that *OTUD7B* regulation, probably owing to miR-1180 downregulation, may negatively regulate the NF-κB-Lin28 axis which in turn triggers Let-7 microRNA-mediated caspase-3 downregulation, thereby conferring paclitaxel resistance in TNBCs. These findings suggest that *OTUD7B* may be a useful biomarker for predicting the anti-cancer effectiveness of paclitaxel and could serve as a new drug target for enhancing the canceridal efficiency of paclitaxel against TNBCs.

## INTRODUCTION

Paclitaxel is a tubulin-targeting agents [1] commonly used in currently anti-cancer therapies. In the updated National Comprehensive Cancer Network (NCCN) guidelines (www.nccn.org/patients), paclitaxel-based adjuvant chemotherapy is still recommended as a first-line regiment for treating patients with triple-negative breast cancer (TNBC). Paclitaxel targets microtubules to interfere with the mitotic spindle, resulting in cell cycle arrest and ultimately apoptosis [2]. Although paclitaxel eliminates most tumor cells, the mechanisms causing resistance in residual cancer cells are unclear. Drug resistance is a crucial problem in cancer therapy that impacts mortality rates [3]. Recently, the combination of paclitaxel with carboplatin was shown to significantly increase the proportion of 315 TNBC patients achieving a pathological complete response (pCR): this therapy produced a pCR rate of 59% compared with 38% pCR in patients who did not receive carboplatin [4]. Moreover, a randomized phase II trial that enrolled 443 patients with stage II to III TNBC receiving standard paclitaxel therapy with or without carboplatin found that the combination resulted in a significantly higher pCR rate (54% vs. 41%) [5]. These findings indicate that the majority of TNBC patients are likely insensitive to paclitaxel treatment. Since patients suffered from chemotherapy, identification of useful biomarkers for predicting pCR in TNBC patients receiving paclitaxel-based chemotherapies is urgently needed.

Recent studies have demonstrated that an aberrant expression of β-tubulin subtype *TUBB3* due to amino acid alteration at the paclitaxel-binding site predicts a poor response to paclitaxel-based chemotherapy and unfavorable prognosis in patients with metastatic gastric cancer [6], ovarian cancer [7] and non-small cell lung cancer [8, 9]. Although *TUBB3* gene expression appeared to be an independent factor for breast cancer patients with luminal A/B subtypes, a Cox regression analysis of recurrence-free survival probability revealed that *TUBB3* was not a useful prognostic factor for patients with HER2-enriched and basal-like (>75% triple-negative) breast cancers [10]. This result indicates that *TUBB3* expression may be not a good biomarker for predicting paclitaxel response in these HER2-positive and triple-negative breast cancer patients. Even though alteration of β-tubulin subtypes may affect the anti-cancer effectiveness of paclitaxel, the discovery of new biomarkers other than *TUBB3* to predict clinical outcomes in patients with HER2-positive and triple-negative breast cancers prior to receiving paclitaxel-based chemotherapy is likely valuable.

The 16 human Ovarian Tumour (OTU) family deubiquitinases (DUBs) are key regulators of the ubiquitin (Ub) code. Small OTU DUBs of the OTUD and OTUB subfamilies employ distinct mechanisms to achieve linkage specificity but the physiological roles of most members are unclear [11–13]. Accumulated evidence indicated that *OTUD7B* regulates inflammation and NF-κB signaling, T-cell activation and epidermal growth factor receptor (EGFR) trafficking [14–16]. IHere we find that *OTUD7B* were predominantly upregulated in paclitaxel-resistant MDA-MB436 TNBC cells after paclitaxel treatment and highly correlated with IC_50_ concentrations of paclitaxel in tested TNBC cell lines. Conversely, *OTUD7B* was dramatically downregulated in paclitaxel-sensitive HCC38 TNBC cells following treatment with paclitaxel. Intriguingly, compared to other OTUDs, *OTUD7B* transcript in tumors was significantly higher than that of normal tissues derived from patients with breast cancer. Moreover, *OTUD7B* upregulation was appeared to predict an increased risk for cancer recurrence/metastasis in breast cancer patients who may exhibit a poor pathologic complete response to paclitaxel chemotherapy. Finally, the *in silico* analysis showed that the loss of miR-1180 and the inhibition of inflammation-related pathways may be associated with *OTUD7B*-related mechanisms underlying paclitaxel resistance in TNBCs.

## RESULTS

### *OTUD7B* is predominantly upregulated following paclitaxel treatment in TNBC cells

**Figure 1:**
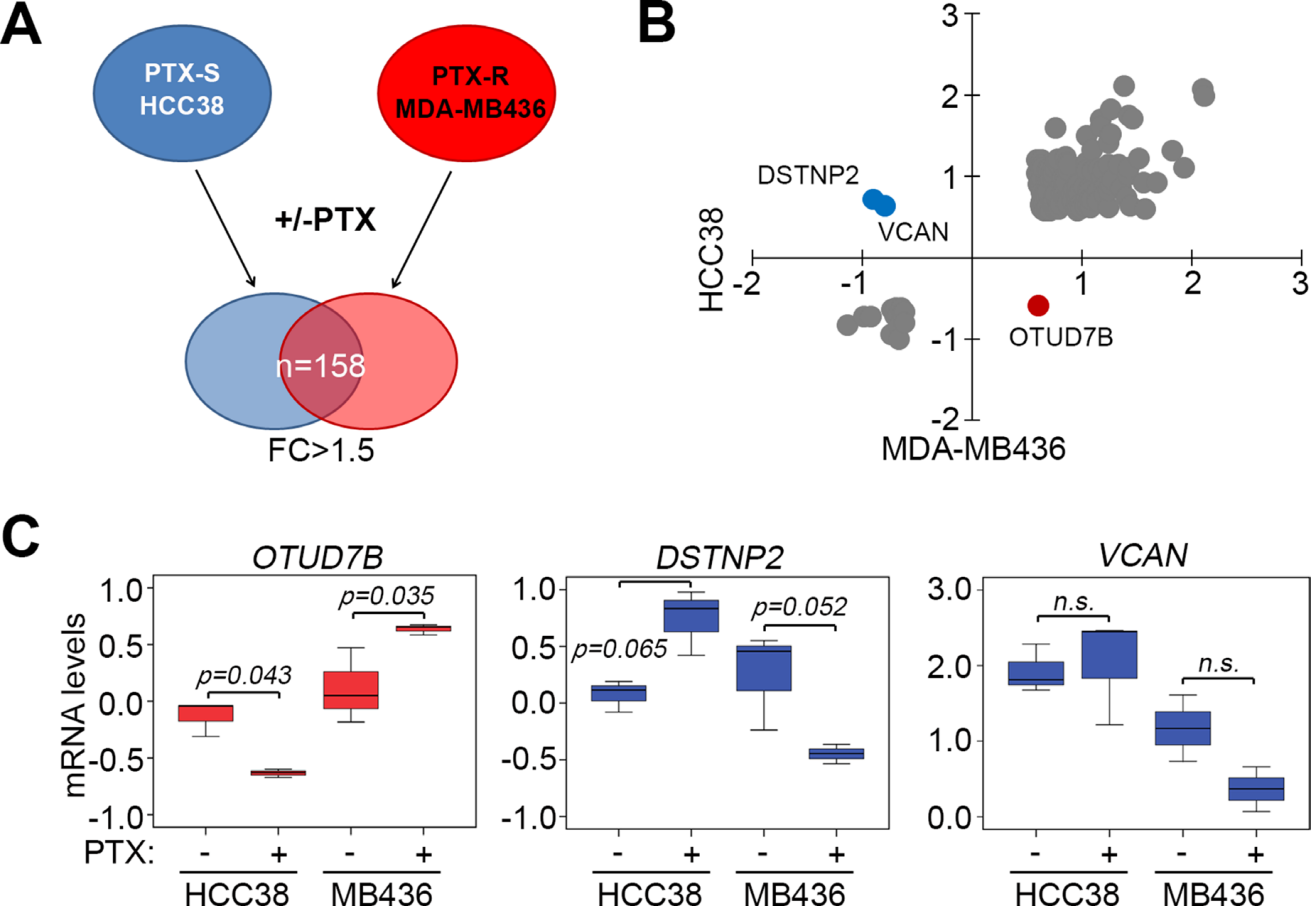
*OTUD7B* upregulation and *DSTNP*/*VCAN* downregulation predict a poor response to paclitaxel treatment in TNBC cells (**A**) Flowchart of the procedure for identifying consensus genes with 1.5-fold change (FC) post-treatment with paclitaxel (PTX) at a concentration of 10 × IC_50_ for 24 hours in PTX-sensitive (PTX-S) HCC38 cells and PTX-resistant (PTX-R) MDA-MB436 cells. (**B**) Dotplot of mRNA levels (log_2_) of 158 consensus genes identified using the strategy shown in A. (**C**) mRNA levels of *OTUD7B*, *DSTNP*2 and *VCAN* in HCC38 and MDA-MB436 cells following treatment without or with paclitaxel at 10 × IC_50_ for 24 hours. Data from three independent experiments are shown as medians ± SD. Statistical differences were analyzed using one-way ANOVA with Tukey’s test.

To identify the molecules that are responsible for paclitaxel resistance in TNBCs, we analyzed transcriptional profiles of paclitaxel-sensitive HCC38 and paclitaxel-resistant MDA-MB436 with or without paclitaxel treatment for 24 hours at their respective 10-fold IC_50_ concentrations determined previously [17]. Then, we dissected 158 consensus genes with 1.5-fold changes after paclitaxel treatment in HCC38 and MDA-MB436 cells (Figure 1A, Supplementary Table 1). Our data showed that *OTUD7B* was upregulated but *DSTNP2*, destrin, actin depolymerizing factor pseudogene 2, and *VCAN*, a member of the aggrecan/versican proteoglycan family, were downregulated in MDA-MB436 cells (Figure 1B). An inversed effect on mRNA expression was detected in HCC38 cells (Figure 1B). However, mRNA levels from three independent experiments indicated that the transcriptional activity of *OTUD7B* was altered more dramatically upon paclitaxel treatment in both HCC38 and MDA-MB436 cells (Figure 1C) compared to *DSTNP2* and *VCAN*.

**Figure 2:**
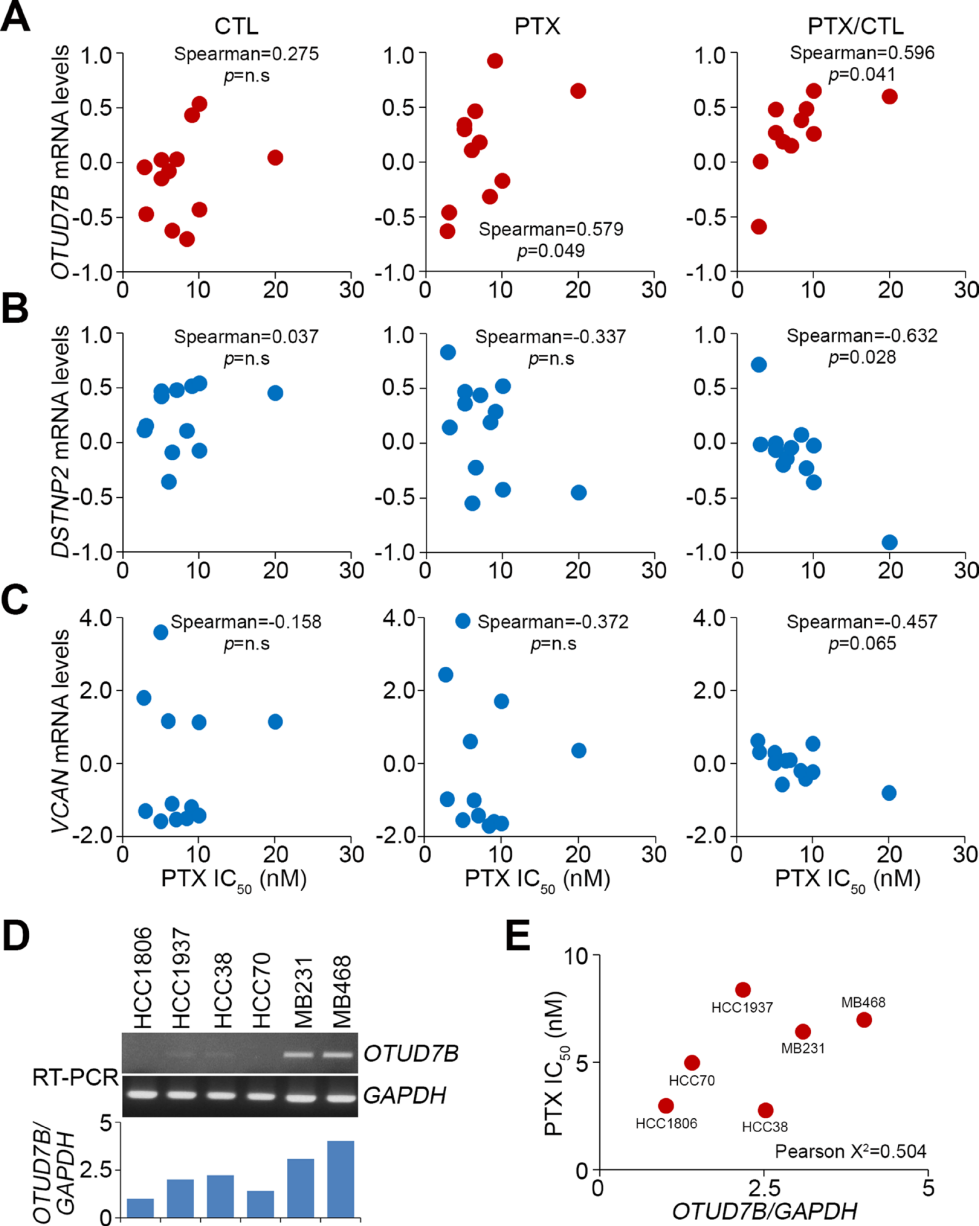
*OTUD7B* and *DSTNP*/*VCAN* positively and negatively correlate, respectively, with PTX IC_50_ concentrations in a panel of TNBC cell lines (**A**–**C**) Correlations between *OTUD7B*, *DSTNP* and *VCAN* mRNA level and PTX IC_50_ concentration in tested TNBC cell lines. Correlations were assessed using Spearman’s test. The symbol “n.s” denotes non-significant results. Each dot in the dotplot indicates the median of mRNA levels from three independent experiments. (**D**) RT-PCR analysis for *OTUD7B* and *GAPDH* transcripts in TNBC cell lines (*top*). The levels of *OTUD7B* transcript were normalized by comparing with the respective *GAPDH* level in the tested cell lines and shown as ratios (*bottom*). (**E**) Correlates between the normalized *OTUD7B* levels and PTX IC_50_ concentrations in a panel of TNBC cell lines. Pearson’s correlation test was used to evaluate the statistical significance.

Furthermore, we analyzed correlation between the IC_50_ concentrations of paclitaxel and the mRNA levels of *OTUD7B*, *DSTNP2* and *VCAN* in a panel of TNBC cell lines BT-20, BT-549, HCC-1143, HCC-1806, HCC-1937, HCC-1954, HCC-30, HCC-70, Hs578T, MDA-MB-231, MDA-MB-436 and MDA-MB-468 after treatment with or without paclitaxel at the respective 10-fold IC_50_ concentrations. The data showed that correlations between the IC_50_ concentrations of paclitaxel and *OTUD7B* mRNA levels were marginal in the paclitaxel-untreated cells and significantly after treatment with paclitaxel in the tested TNBC cells (Figure 2A). Compared to untreated control cells, the correlation of *OTUD7B* mRNA levels with IC_50_ concentrations of paclitaxel appeared to be more significant in the tested TNBC cells (Figure 2A). In contrast, in both paclitaxel-treated and -untreated cells, IC_50_ concentrations of paclitaxel were inversely correlated with *DSTNP2* (except in control cells) and *VCAN* mRNA levels (Figure 2B and 2C). In comparison with control cells, the mRNA levels of *DSTNP2* and *VCAN* were determined respectively to significantly and marginally correlate with the IC_50_ concentrations of paclitaxel in the detected TNBC cells (Figure 2B and 2C). To validate these findings, we performed reverse transcription PCR (RT-PCR) experiment in order to determining the *OTUD7B* mRNA levels in a panel of TNBC cells (Figure 2D). Our data showed that the levels of *OTUD7B* transcript causally associated with the PTX IC_50_ concentrations in the detected TNBC cells (Figure 2E). Based on these findings, we subsequently focused on estimating the prognostic significance of *OTUD7B* in predicting the effectiveness of paclitaxel on clinical patients with breast cancer.

### *OTUD7B* upregulation correlates with tumorigenesis and poor recurrence-free survival rates in clinical patients with TNBCs

**Figure 3:**
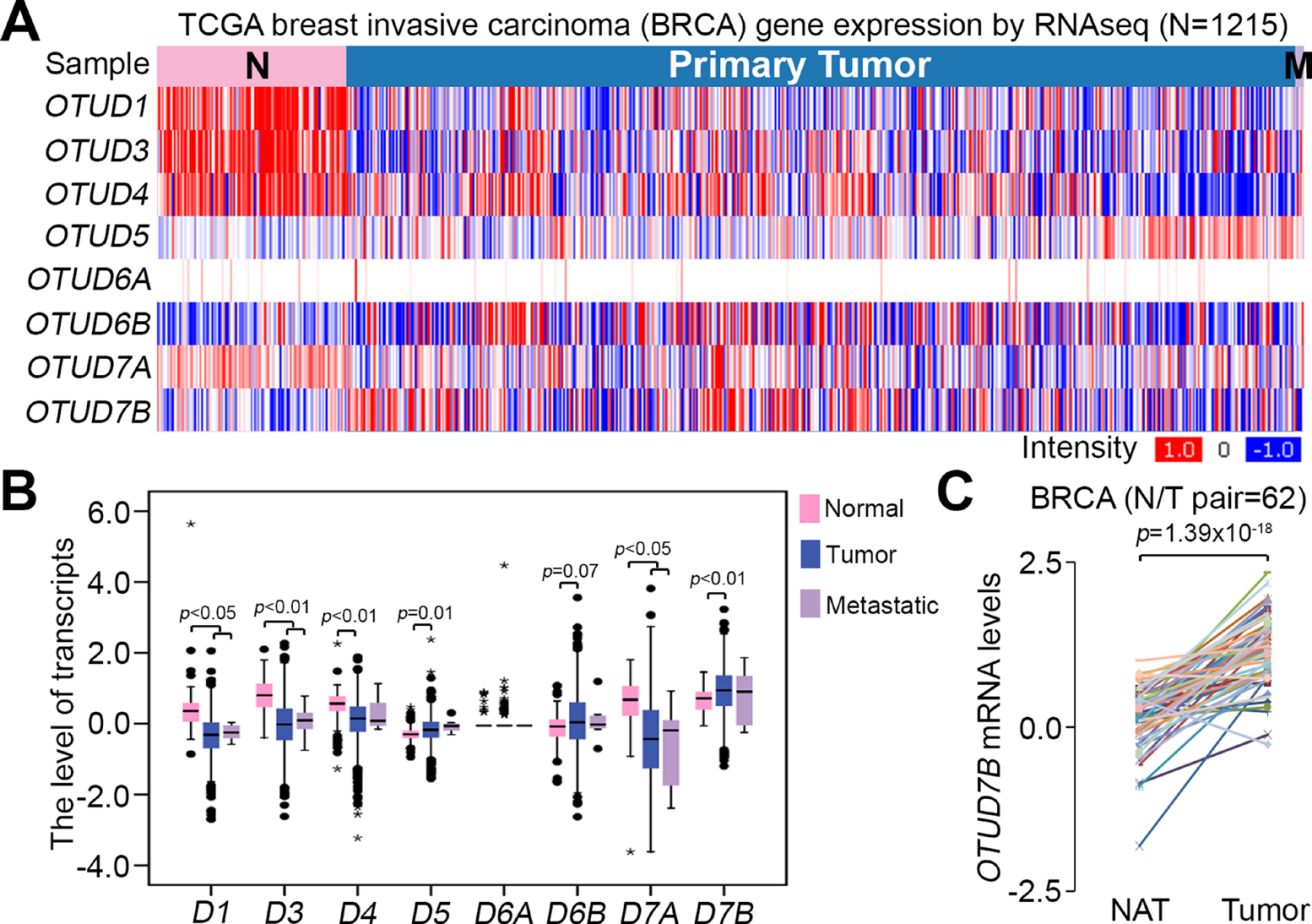
*OTUD7B* expression is upregulated in primary tumors compared to normal tissues derived from patients with breast cancer (**A**) Heatmap for the transcription profile of *OTUD* subtypes in normal tissues, primary tumors and metastatic tumors derived from patients with breast invasive carcinoma (BRCA) using the TCGA database. (**B**) Boxplot of mRNA levels of *OTUD* subtypes in defined normal tissues (*n* = 113), primary tumors (*n* = 1061) and metastatic tumors (*n* = 7) from A. Data were analyzed by One-way ANOVA using Turkey’s test. (**C**) *OTUD7B* expression in paired normal adjacent tissues (NAT) and primary tumors derived from patients with BRCA. Data were analyzed using paired *t*-tests.

To examine the association of *OTUD7B* expression with breast cancer development, we next analyzed the transcriptional profile of *OTUD7B*, as well as other OTUD subtypes, in TCGA breast invasive carcinoma (BRCA). The data showed that primary tumors expressed *OTUD7B* more abundantly than normal mammary epithelial tissues derived from patients with BRCA (Figure 3A). Moreover, among OTUD subtypes, the level of *OTUD7B* mRNA was detected to be much abundant in primary tumors compared to normal tissues derived from patients with breast cancer (Figure 3B). Accordingly, our data showed that the mRNA levels of *OTUD7B* in primary tumors are significantly higher than in normal adjacent tissues derived from BRCA patients (Figure 3C). Similar findings were also observed in paired normal adjacent tissues and primary tumors derived from patients with basal-like breast cancers, approximately 75% of which were TNBCs (Supplementary Figure 1).

**Figure 4:**
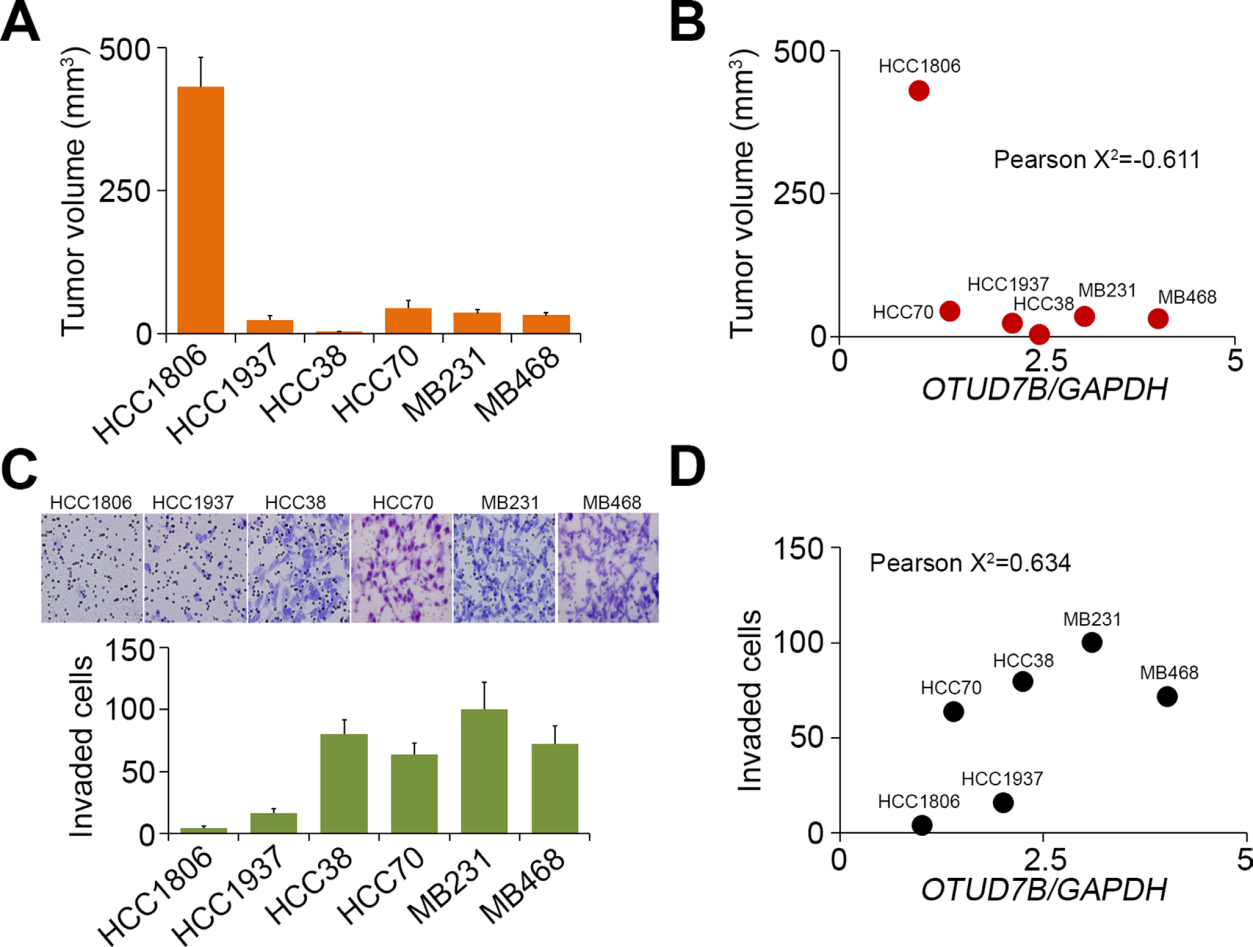
The correlations of *OTUD7B* transcriptional levels with the tumor growth, invasion and lung metastatic colonization abilities of TNBC cell lines (**A**, **B**) Mice (*n* = 5) were subcutaneously implanted with breast cancer cell lines as indicated for 4 weeks. Tumor volume was measured from tumor-bearing mice and presented as mean ± SD (A). The correlation between *OTUD7B* transcriptional levels and the mean of tumor volume in each group was shown as dotplot (B). (**C**, **D**) The invaded cells in the invasion assay were stained with Giemsa regent (C, top). The data from triplicate experiments were presented as mean ± SD (C, bottom). The correlation between the normalized *OTUD7B* levels and invaded cell numbers of the tested cell lines was shown by dotplot (D). In (B, D), Pearson’s correlation test was used to evaluate the statistical significance.

We next examined the *in vivo* tumor growth of TNBC cell lines HCC1806, HCC1937, HCC38, HCC70, MDA-MB231 and MDA-MB468 through the subcutaneous implantation into NOSCID mice for 4 weeks (Figure 4A). The data showed that tumor volumes of the tested TNBC cells post-implantation for 4 weeks are negatively correlated with *OTUD7B* mRNA levels (Figure 4B). Moreover, we detected the *in vitro* invasion ability of several TNBC cell lines HCC1806, HCC1937, HCC38 and MDA-MB231 by using trans-well cultivation (Figure 4C). Our results revealed that *OTUD7B* mRNA levels causally correlated with invasive ability of the tested TNBC cell lines (Figure 4D).

**Figure 5:**
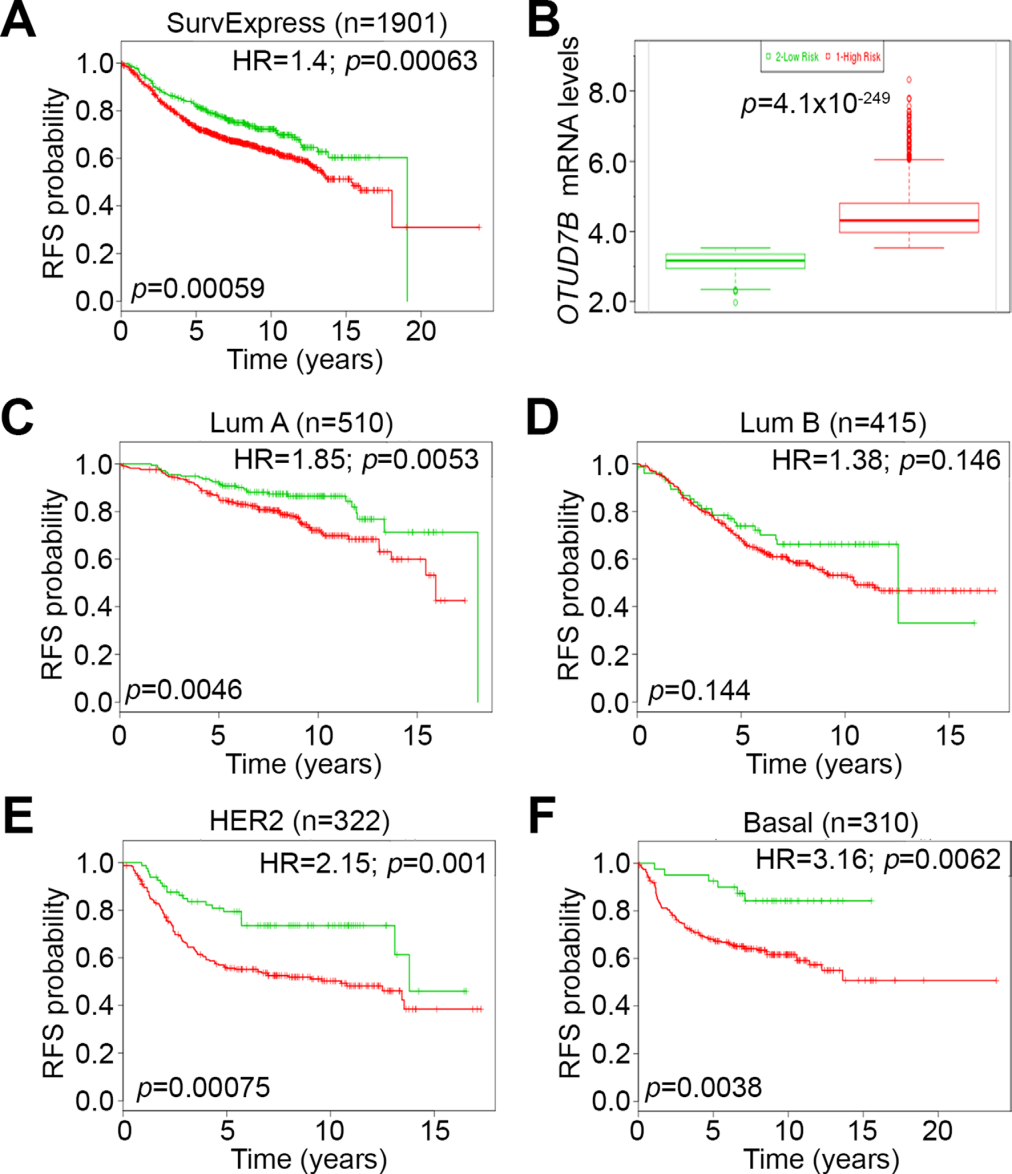
*OTUD7B* upregulation is associated with poor recurrence-free survival rates in breast cancer patients (**A**) Kaplan-Meier analysis of recurrence-free survival (RFS) probability according to *OTUD7B* expression in breast cancer patients, performed using the SurvExpress database. HR denotes hazard ratio. (**B**) Boxplot of *OTUD7B* mRNA levels in low (green) and high (red)-risk cohorts in A. (**C–F**) Kaplan-Meier analysis of RFS probability according to *OTUD7B* expression in breast cancer patients with luminal A (Lum A), luminal B (Lum B), HER2 and basal-like (Basal) subtypes.

Because chemotherapeutic efficacy is frequently assessed by recurrence-free survival (RFS) probability, we further estimated the prognostic significance of *OTUD7B* in predicting RFS rates in breast cancer patients. Using the SurvExpress database, we found that high-levels of *OTUD7B* mRNA expression significantly correlated with poor RFS rates, with a hazard ratio (HR) of 1.4 in 1901 breast cancer patients (Figure 5A). *OTUD7B* mRNA levels in the high-risk cohort were significantly upregulated compared to the low-risk cohort among enrolled breast cancer patients (Figure 5B). Similar results were also observed in the another cohort with 1574 breast cancer patients in SurvExpress database (Supplementary Figure 2). To investigate the predictive relevance among patients with different breast cancer subtypes, we performed further Kaplan-Meier analysis to compare RFS probability based on *OTUD7B* expression for patients represented in Figure 5A with luminal A/B, HER2-positive and basal-like breast cancers (Figure 5C–5F). Our data showed that *OTUD7B* upregulation was significantly associates with a poor RFS rate in patients with HER2-positive and basal-like breast cancers (Figure 5E and 5F); the worst HR was observed in patients with basal-like breast cancer compared to other subtypes (Figure 5F). These findings might implicate that *OTUD7B* severs as a useful biomarker to predict poor prognosis in patients with estrogen receptor-negative breast cancers.

### *OTUD7B* upregulation predicts a poor response to paclitaxel-based chemotherapy in breast cancer patients

**Figure 6:**
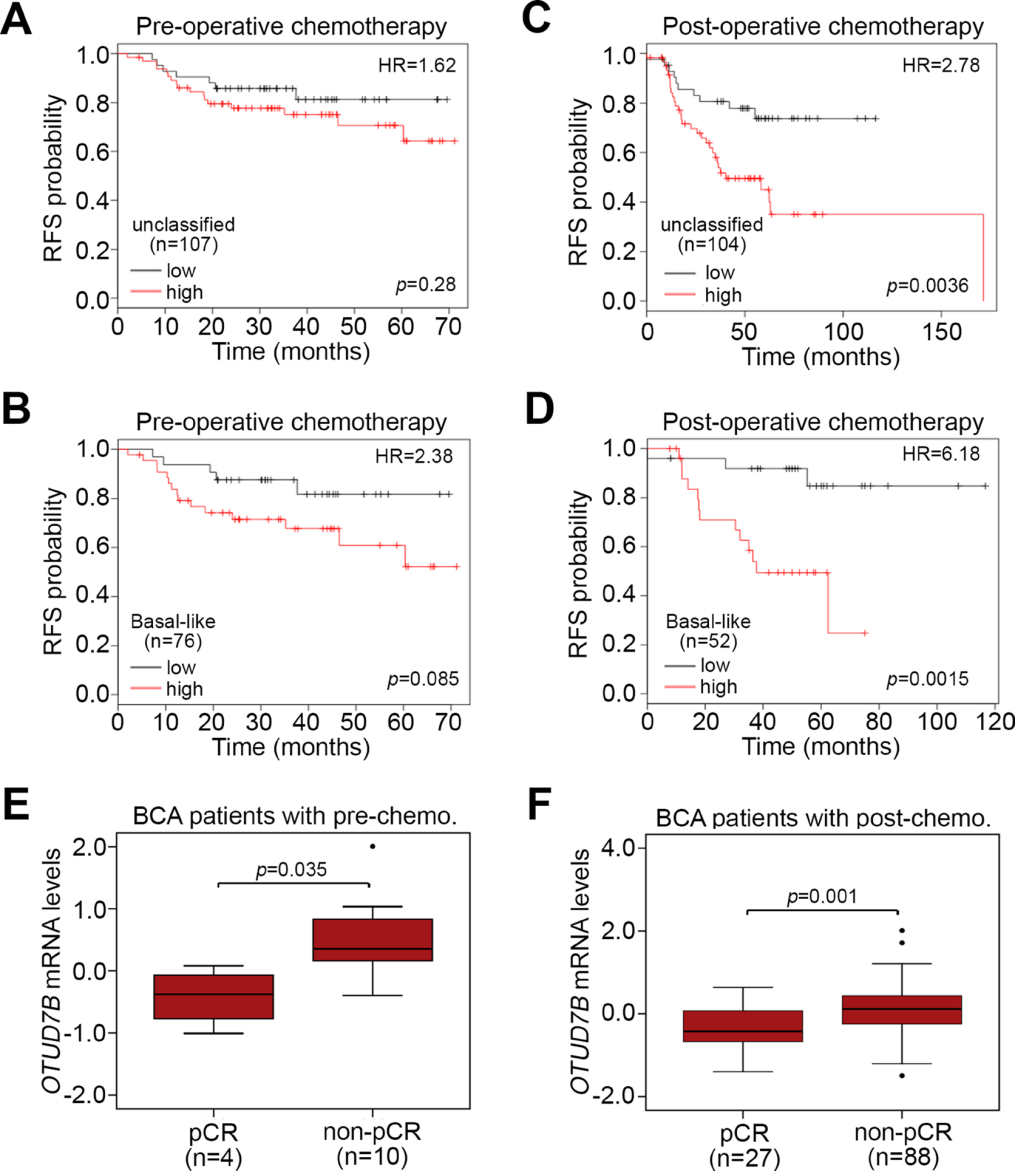
*OTUD7B* upregulation predicts a poor response to paclitaxel chemotherapy in breast cancer patients (**A, B**) Kaplan-Meier analysis of RFS probability according to *OTUD7B* expression in patients who receive pre-operative paclitaxel-based chemotherapy with unclassified (A) or basal-like (B) breast cancer. (**C, D**) Kaplan-Meier analysis of RFS probability according to *OTUD7B* expression in patients who receive post-operative paclitaxel-based chemotherapy with unclassified (C) or basal-like (D) breast cancer. In A-D, Kaplan-Meier analyses were performed using the K-M Plotter database and HR denotes hazard ratio. (**E, F**) Boxplot of OTUD7B mRNA levels in tumor biopsy derived from BCA patients who received pre-operative paclitaxel-based chemotherapy (pre-chemo.) (E) or pre-operative paclitaxel-based chemotherapy (post-chemo.) (F). pCR denotes pathological complete response. Data were analyzed using a non-parametric Mann-Whitney *U*-test.

We next evaluated the prognostic significance of *OTUD7B* in breast cancer patients who received paclitaxel-based chemotherapy. Using the K-M Plotter database, we found that *OTUD7B* upregulation reflected an unfavorable prognosis in terms of RFS probability in patients receiving pre-operative paclitaxel chemotherapy with unclassified (Figure 6A) and basal-like (Figure 6B) breast cancer. On the other hand, *OTUD7B* upregulation more significantly (*p <* 0.05) predicted a poor RFS probability in patients receiving post-operative paclitaxel chemotherapy with unclassified (Figure 6C) and basal-like (Figure 6D) breast cancer. Similar views were also observed in K-M analysis under the condition of distant metastasis-free survival probability using K-M Plotter database against patients receiving post-operative paclitaxel chemotherapy with unclassified (Supplementary Figure 3A) and basal-like (Supplementary Figure 3B) breast cancer. In comparison, we analyzed the transcriptional profile of *OTUD7B* in breast tumors derived from breast cancer patients receiving pre-operative paclitaxel-based chemotherapy. The data revealed that *OTUD7B* mRNA levels in breast tumors derived from patients with non-pathological complete response (pCR) were significantly higher than in patients with (Figure 6E). Similarly, among patients receiving post-operative paclitaxel-based chemotherapy, our results showed that *OTUD7B* mRNA levels in breast tumors derived from patients with non-pCR were predominantly elevated compared to those of patients with pCR (Figure 6F).

### *OTUD7B* upregulation is associates with inhibition of inflammation-related pathways and activation of *Let-7*-mediated post-transcriptional regulation in paclitaxel-resistant TNBC cells

**Figure 7:**
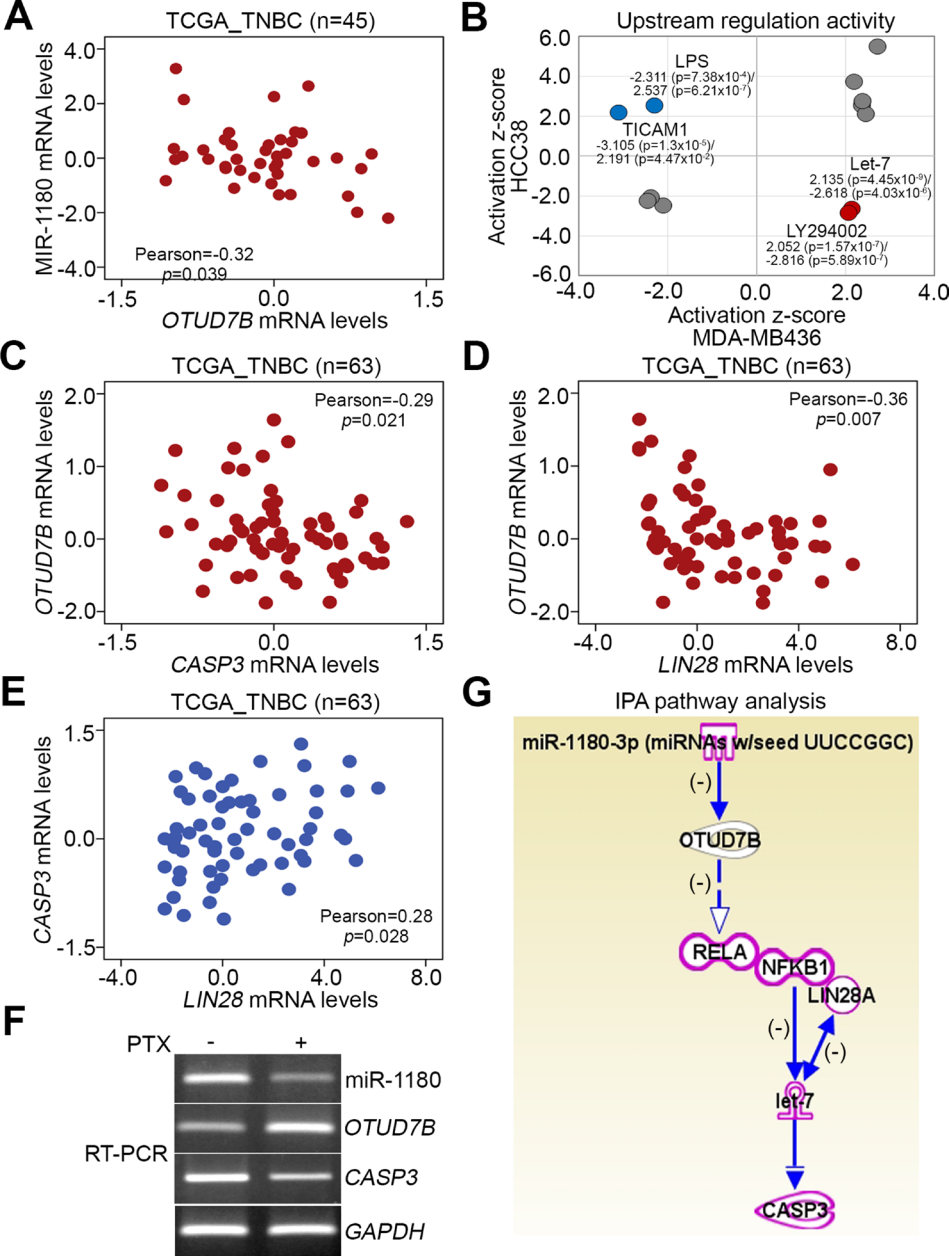
Possible mechanisms underlying paclitaxel resistance in TNBC cells (**A**) Correlation between miR-1180 and *OTUD7B* mRNA levels in primary tumors derived from patients with TNBC using the TCGA database. Pearson’s correlation test was used to estimate the correlation. (**B**) *In silico* analysis of consensus upstream regulators that were possibly activated or inhibited after paclitaxel treatment at 10 × IC_50_ for 24 hours in HCC38 and MDA-MB436 cells, performed using Ingenuity Pathway Analysis software. (**C**–**E**) Correlations among *OTUD7B*, CASP3 and LIN28 mRNA levels in clinical tissues derived from patients with TNBC using TCGA database. In A, C, D and E, Pearson’s correlation test was used to estimate the statistical association. (**F**) RT-PCR analysis for primary miR-1180, OTUD7B, CASP3 and GAPDH transcripts derived from MDA-MB468 cells treated without or with PTX at 50 nM for 6 hours. (**G**) The computational simulation of pathway axis consisted with miR-1180, OTUD7B, NF-kB/LIN28, Let-7 and CASP3 by Ingenuity Pathway Analysis (IPA) software.

To ascertain the possible mechanism for *OTUD7B* upregulation-related paclitaxel resistance in TNBCs, we next used the TCGA database to analyze the transcriptional profile of *OTUD7B* and microRNA (miRNA)-1180 which has been shown to trigger post-transcriptional downregulation of *OTUD7B* [18] in primary tumors derived from patients with TNBC. We found that mRNA expression levels of *OTUD7B* and miR-1180 were inversely related in tested tumor tissues (Figure 7A). Moreover, we performed an *in silico* analysis using Ingenuity Pathway Analysis (IPA) software to computationally simulate possible activated or inhibited upstream regulators either in paclitaxel-sensitive HCC38 cells or paclitaxel-resistant MDA-MB436 cells after treatment with paclitaxel at the 10-folds IC_50_ concentrations for 24 hours. Our data showed that activation of Let-7 microRNA and LY294002, a pharmaceutical inhibitor for phosphatidylinositol 3-kinase (PI3K), was significantly predicted; conversely, inhibition of lipopolysaccharide (LPS) and toll-like receptor adaptor molecule 1 (TICAM1)-related signaling cascades was strongly predicted in MDA-MB436 cells following treatment with paclitaxel (Figure 7B, Supplementary Table 2). The inverse activity of these upstream regulators was computationally simulated in HCC38 cells (Figure 7B). Using the IPA database, we identified several Let-7-targeting genes in HCC38 and MDA-MB436 cells upon paclitaxel stimulation (Supplementary Figure 4A and 4B).

Since Let-7 microRNA has been shown to inhibit *CASP3* expression, thereby promoting chemoresistance [19], and be negatively regulated by the NF-kB-Lin28 axis in breast cancer cells [20], we next analyzed the transcriptional profiles of *OTUD7B*, *CASP3* and *LIN28* in TNBC tissues. The data showed that *OTUD7B* expression was inversely correlated with *CASP3* and *LIN28* (Figure 7C and 7D), whereas mRNA levels of *CASP3* and *LIN28* (Figure 7E) were positively correlated in TNBC tissues. Moreover, RT-PCR analysis revealed that the mRNA levels of miR1180 and *CASP3* are downregulated but *OTUD7B* transcript is upregulated upon PTX treatment in MDA-MB468 cells (Figure 7F). By using IPA software, the transcriptional inhibition of miR1180 on *OTUD7B* and the negative regulation of *OTUD7B* towards the NF-kB-Lin28 axis-inhibiting let-7 function on the transcriptional repression of *CASP3* were computationally simulated (Figure 7G).

## DISCUSSION

Resistance is a significant limitation to the effectiveness of cancer therapies. Here, we show that *OTUD7B* is upregulated in paclitaxel-resistant MDA-MB436 cells but downregulated in paclitaxel-sensitive HCC-38 cells after treatment with paclitaxel at 10-fold IC_50_ concentrations for 24 hours. Moreover, *OTUD7B* upregulation significantly predicts an unfavorable risk for cancer recurrence/metastasis and is associated with a poor response to paclitaxel-based chemotherapy in breast cancer patients. The molecular mechanism underlying *OTUD7B* upregulation-related paclitaxel resistance is likely associated with the downregulation of *OTUD7B*-targeting miR-1180 and LPS/PI3K-related signaling cascades and as well as the induction of miR-Let-7-mediated post-transcriptional inhibition of multiple genes in TNBC cells.

Previous studies have demonstrated that *OTUD7B* is a negative regulator for the NF-κB-related pathway through deubiquitination of TNF receptor-associated factor 3 (TRAF3) in mucosal immunity against infections [15]. Here, we find that paclitaxel-resistant MDA-MB436 cells suppress the LPS- and toll-like receptor adaptor molecule 1 (TICAM1)-related signaling pathways, which ultimately converge on the activation of NF-κB [21, 22]. Activation of the NF-kB-related pathway is known to be critical for mediating the cellular response to oxidative stress and inflammation [23–25]. Increased oxidative stress has been shown previously to affect potential paclitaxel cancercidal effectiveness in ovarian cancer [26] . Based on these findings, we conclude that negative regulation of the NF-κB-related pathway by upregulated *OTUD7B* is a key mechanism underlying paclitaxel resistance in TNBCs. In contrast, the NF-κB-related pathway was known to control cell growth by regulating the cell cycle machinery such as cyclins D1 [27, 28] which plays as a key element in mammary gland development and breast carcinogenesis [29]. Here we show that the *OUTD7B* upregulation inversely correlates with the *in vivo* tumor growth in a panel of TNBC cell lines even though the increased levels of *OUTD7B* transcript are extensively found in primary tumors in comparison with normal tissues derived from patients with breast cancer. Therefore, *OTUD7B* upregulation may suppress tumor growth via negatively regulating NF-κB-related pathway in TNBCs.

The PI3K/Akt pathway plays important roles in tumorigenesis and drug resistance in human cancers [30, 31]. A previous study has demonstrated that LY294002, a specific inhibitor of the PI3K/Akt pathway, induces resistant cells to become more sensitive to paclitaxel or docetaxel treatment in prostate cancer cells [30]. In contrast, inhibition of the LPS-triggered inflammation response appeared to suppress oxidative stress-mediated activation of the PI3K/Akt-NF-κB signaling axis in macrophages [32]. Interestingly, a recent report demonstrated that blockage of LPS-induced inflammatory responses inhibits the activity of the PI3K/Akt-NF-κB pathway but induces activation of the Nrf2 pathway [33], which is thought to be correlated with mechanism for chemoresistance in many cancer types [34–39], including breast cancer [40, 41]. In our simulation, the pharmaceutical inhibitory effect of LY294002 on PI3K/Akt pathway was activated upon paclitaxel stimulation in paclitaxel-resistant TNBC cells. Again, this finding supports the hypothesis that induction of *OTUD7B*-mediated inactivation of the NF-κB-related pathway plays a pivotal role in the mechanism for developing paclitaxel resistance in paclitaxel-resistant TNBC cells.

Previously, we have shown that induction of Let-7 microRNA inhibits the expression of caspase-3, a master caspase in mediating cell apoptosis, and thereby confers multidrug resistance in breast cancer cells [19]. Similar findings have also been reported in other cancer types [42]. Here, we show that Let-7 microRNA is computationally predicted to be highly active in paclitaxel-resistant TNBC cells following paclitaxel treatment. Since our previous report showed that caspase-3 downregulation is predominant in TNBCs with chemoresistance against paclitaxel-based chemotherapy [19], we believed that induction of Let-7 microRNA might be critical for protecting paclitaxel-resistant TNBC cells from paclitaxel-caused cell apoptosis by repressing caspase-3 expression. Induction of the inflammatory response mediated by NF-κB appeared to directly activate Lin28 transcription and rapidly reduce Let-7 microRNA levels, thereby promoting breast cancer tumor growth [20]. It has been known that Lin28 encodes RNA-binding protein that binds to Let-7 pre-microRNA and blocks the production of mature Let-7 microRNA [43, 44]. In contrast, activation of the NF-κB-related pathway due to increased oxidative stress was shown to enhance tumor proliferation but reduce resistance to paclitaxel treatment [26]. Here, we show that *OTUD7B* expression inversely correlates with both *LIN28* and *CASP3* expression in clinical tissues derived from patients with TNBCs. These results suggest that *OTUD7B* upregulation likely inactivates the NF-κB-Lin28 axis, which leads to the activation of Let-7 microRNA-mediated caspase-3 downregulation and eventually results in poor response to paclitaxel-induced cell apoptosis in paclitaxel-resistant TNBC cells.

In conclusion, *OTUD7B* upregulation is most likely due to miR-1180 downregulation and that this upregulation protects paclitaxel-resistant TNBC cells from paclitaxel-induced cell death by inhibiting NF-κB activity and thereby restraining Lin28-mediated suppression of maturation/activation of Let-7 microRNA. These effects may ultimately render the apoptotic pathway ineffective due to Let-7-triggered post-transcriptional inhibition of caspase-3 transcript. Our findings not only suggest that *OTUD7B* could be a prognostic biomarker for paclitaxel efficacy but also imply that targeting *OTUD7B* may be a new strategy to enhance the anti-cancer effectiveness of paclitaxel-based chemotherapy against breast cancer, particularly TNBCs.

## MATERIALS AND METHODS

### Cell lines and cell culture condition

Breast cancer cell lines MDA-MB-231 and MDA-MB468 were cultured in Leibovitz’s (L-15) medium (Gibco Life Technologies, Grand Island, NY, USA) supplemented with 10% foetal bovine serum (FBS, Invitrogen) and incubated at 37°C with free gas exchange with atmospheric air. Breast cancer cell lines HCC1806, HCC1937, HCC38 and HCC70 were cultured in RPMI-1640 medium (Gibco Life Technologies) with 10% FBS and incubated at 37°C with 5% CO_2_. BT-20 and Hs578t cells were cultured in Eagle’s Minimum Essential Medium (EMEM) and DMEM, respectively, with 10% FBS and incubated at 37°C with 5% CO_2_. All cell lines were obtained from American Type Culture Collection (ATCC). All cells were routinely authenticated on the basis of short tandem repeat (STR) analysis, morphologic and growth characteristics and mycoplasma detection.

### Reverse transcription PCR (RT-PCR)

Total RNA was extracted from cells using TRIzol extraction kit (Invitrogen). Aliquots (5 μg) of total RNA were treated with M-MLV reverse transcriptase (Invitrogen) and then amplified with Taq-polymerase (Protech) using paired primers (for primary mR-1180, forward-CTGGTGCCCACCTCAGAGACGG and reverse-CACAGCCACCAGGCTGAGCATG; for *OTUD7B*, forward-GAAGGAGAAGTCAAAGCGAGATCG and reverse-GCATCACCTCCTGGCTATACTTGC; for *CASP3*, forward-CATGGAAGCGAATCAATGGACT and reverse-CTGTACCAGACCGAGATGTCA; for *GAPDH*, forward-AGGTCGGAGTCAACGGATTTG and reverse-GTGATGGCATGGACTGTGGTC).

### *In vitro* invasion assay

For invasion assay, Boyden chambers (Neuro Probe, Inc., USA) were used according to the manufacturer’s protocol. Briefly, polycarbonate membrane (8 μm pore size, 25 × 80 mm, Neuro Probe, Inc., USA) was pre-coated with 10 μg of human fibronectin (Sigma, MO, USA) on the lower side and matrigel on the upper side. Cells (1.5 × 10^4^) were plated in the top chamber in 50 μl of starvation medium (0.1% FBS) containing drugs or DMSO. After 16 h, stationary cells were removed from the top side of the membrane, whereas migrated cells in the bottom side of the membrane was fixed in 100% methanol and stained with 10% Giemsa’s solution (Merck, Germany) for 1 hr. Invaded cells were counted under the light microscope (400x, ten random fields from each well). All experiments were performed in triplicates.

### Microarray data processing

Transcriptional profiling results for HCC38 and MDA-MB436 cells treated with or without paclitaxel at 10× IC_50_ concentrations were obtained from GEO DataSets (accession no. GSE50832) on the NCBI website. Microarray data and related clinical data for GEO DataSet GSE22513 were also downloaded from NCBI website. Affymetrix DAT files were processed using the Affymetrix Gene Chip Operating System (GCOS) to generate .CEL files. Raw intensities in the .CEL files were normalized by robust multi-chip analysis (RMA), and fold-change analysis was performed using GeneSpring GX11 (Agilent Technologies). Relative mRNA expression levels were normalized by their median and presented as log_2_ values.

### *In silico* analysis

Genes with a 1.5-fold-change threshold relative to control cells in HCC38 and MDA-MB436 cells after paclitaxel treatment were uploaded to the Ingenuity Pathway Analysis (IPA) website (Ingenuity Systems, www.ingenuity.com). Results of computational predictions for the activation or inhibition status of upstream regulators were then output as a text file. Consensus upstream regulators with significant z-scores from *in silico* analysis of paclitaxel-treated HCC38 and MDA-MB436 cells were analyzed in a PivotTable report and plotted as a dotplot in Microsoft Excel.

### Animal studies

NOD/SCID mice were obtained from National Laboratory Animal Center in Taiwan and were maintained in compliance with the institutional guideline. All animal procedures were approved by the Institutional Animal Care and Use Committee at Academia Sinica, Taiwan. For the *in vivo* tumor growth, cells (5 × 10^5^) suspended in 50 μL PBS were subcutaneously inoculated into the lateral dorsal skin of each mouse. Mice were humanely killed at the end of experiments and lungs were obtained for histological analysis.

### Statistical analyses

SPSS 17.0 software (Informer Technologies, Roseau, Dominica) was used to analyze statistical significance. Paired *t*-tests were utilized to compare *OTUD7B* gene expression in cancer tissues and corresponding normal tissues. Spearman’s test was performed to estimate the association between *OTUD7B* mRNA and Paclitaxel IC_50_ concentrations in the panel of TNBC cell lines. Pearson’s correlation test was used to evaluate the association between *OTUD7B* and miR-1180 mRNA expression in basal-like breast cancer. Survival probabilities were determined by Kaplan-Meier analysis and log-rank tests. One-way ANOVA with Tukey’s test was used to estimate the difference in mRNA levels of *OTUD7B*, *DSTNP2* and *VCAN* in HCC38 and MDA-MB436 cells after paclitaxel treatment. Mann-Whitney *U*-tests were used to analyze non-parametric data. *P* values < 0.05 in all analyses were considered statistically significant.

## SUPPLEMENTARY MATERIALS FIGURES AND TABLES





## References

[1] Vyas DM, Kadow JF (1995). Paclitaxel: a unique tubulin interacting anticancer agent. Prog Med Chem.

[2] Yusuf RZ, Duan Z, Lamendola DE, Penson RT, Seiden MV (2003). Paclitaxel resistance: molecular mechanisms and pharm-acologic manipulation. Curr Cancer Drug Targets.

[3] Baguley BC (2010). Multiple drug resistance mechanisms in cancer. Mol Biotechnol.

[4] von MG, Schneeweiss A, Loibl S, Salat C, Denkert C, Rezai M, Blohmer JU, Jackisch C, Paepke S, Gerber B, Zahm DM, Kummel S, Eidtmann H (2014). Neoadjuvant carboplatin in patients with triple-negative and HER2-positive early breast cancer (GeparSixto; GBG 66): a randomised phase 2 trial. Lancet Oncol.

[5] Sikov WM, Berry DA, Perou CM, Singh B, Cirrincione CT, Tolaney SM, Kuzma CS, Pluard TJ, Somlo G, Port ER, Golshan M, Bellon JR, Collyar D (2015). Impact of the addition of carboplatin and/or bevacizumab to neoadjuvant once-per-week paclitaxel followed by dose-dense doxorubicin and cyclophosphamide on pathologic complete response rates in stage II to III triple-negative breast cancer: CALGB 40603 (Alliance). J Clin Oncol.

[6] Hwang JE, Hong JY, Kim K, Kim SH, Choi WY, Kim MJ, Jung SH, Shim HJ, Bae WK, Hwang EC, Lee KH, Lee JH, Cho SH (2013). Class III beta-tubulin is a predictive marker for taxane-based chemotherapy in recurrent and metastatic gastric cancer. BMC Cancer.

[7] Roque DM, Buza N, Glasgow M, Bellone S, Bortolomai I, Gasparrini S, Cocco E, Ratner E, Silasi DA, Azodi M, Rutherford TJ, Schwartz PE, Santin AD (2013). Class III beta-tubulin overexpression within the tumor microenvironment is a prognostic biomarker for poor overall survival in ovarian cancer patients treated with neoadjuvant carboplatin/paclitaxel. Clin Exp Metastasis.

[8] Edelman MJ, Schneider CP, Tsai CM, Kim HT, Quoix E, Luft AV, Kaleta R, Mukhopadhyay P, Trifan OC, Whitaker L, Reck M (2013). Randomized phase II study of ixabepilone or paclitaxel plus carboplatin in patients with non-small-cell lung cancer prospectively stratified by beta-3 tubulin status. J Clin Oncol.

[9] Ohashi T, Yoshimasu T, Oura S, Kokawa Y, Kawago M, Hirai Y, Miyasaka M, Aoishi Y, Kiyoi M, Nishiguchi H, Honda M, Okamura Y (2015). Class III Beta-tubulin Expression in Non-small Cell Lung Cancer: A Predictive Factor for Paclitaxel Response. Anticancer Res.

[10] Xu YC, Zhang FC, Li JJ, Dai JQ, Liu Q, Tang L, Ma Y, Xu Q, Lin XL, Fan HB, Wang HX (2015). RRM1, TUBB3, TOP2A, CYP19A1, CYP2D6: Difference between mRNA and protein expression in predicting prognosis of breast cancer patients. Oncol Rep.

[11] Juang YC, Landry MC, Sanches M, Vittal V, Leung CC, Ceccarelli DF, Mateo AR, Pruneda JN, Mao DY, Szilard RK, Orlicky S, Munro M, Brzovic PS (2012). OTUB1 co-opts Lys48-linked ubiquitin recognition to suppress E2 enzyme function. Mol Cell.

[12] Mevissen TE, Hospenthal MK, Geurink PP, Elliott PR, Akutsu M, Arnaudo N, Ekkebus R, Kulathu Y, Wauer T, El OF, Freund SM, Ovaa H, Komander D (2013). OTU deubiquitinases reveal mechanisms of linkage specificity and enable ubiquitin chain restriction analysis. Cell.

[13] Wiener R, Zhang X, Wang T, Wolberger C (2012). The mechanism of OTUB1-mediated inhibition of ubiquitination. Nature.

[14] Hu H, Wang H, Xiao Y, Jin J, Chang JH, Zou Q, Xie X, Cheng X, Sun SC (2016). Otud7b facilitates T cell activation and inflammatory responses by regulating Zap70 ubiquitination. J Exp Med.

[15] Hu H, Brittain GC, Chang JH, Puebla-Osorio N, Jin J, Zal A, Xiao Y, Cheng X, Chang M, Fu YX, Zal T, Zhu C, Sun SC (2013). OTUD7B controls non-canonical NF-kappaB activation through deubiquitination of TRAF3. Nature.

[16] Pareja F, Ferraro DA, Rubin C, Cohen-Dvashi H, Zhang F, Aulmann S, Ben-Chetrit N, Pines G, Navon R, Crosetto N, Kostler W, Carvalho S, Lavi S (2012). Deubiquitination of EGFR by Cezanne-1 contributes to cancer progression. Oncogene.

[17] Dezso Z, Oestreicher J, Weaver A, Santiago S, Agoulnik S, Chow J, Oda Y, Funahashi Y (2014). Gene expression profiling reveals epithelial mesenchymal transition (EMT) genes can selectively differentiate eribulin sensitive breast cancer cells. PLoS.One.

[18] Tan G, Wu L, Tan J, Zhang B, Tai WC, Xiong S, Chen W, Yang J, Li H (2016). MiR-1180 promotes apoptotic resistance to human hepatocellular carcinoma via activation of NF-kappaB signaling pathway. Sci Rep.

[19] Lin YF, Lai TC, Chang CK, Chen CL, Huang MS, Yang CJ, Liu HG, Dong JJ, Chou YA, Teng KH, Chen SH, Tian WT, Jan YH (2013). Targeting the XIAP/caspase-7 complex selectively kills caspase-3-deficient malignancies. J Clin Invest.

[20] Iliopoulos D, Hirsch HA, Struhl K (2009). An epigenetic switch involving NF-kappaB, Lin28, Let-7 MicroRNA, and IL6 links inflammation to cell transformation. Cell.

[21] Cao L, Li R, Chen X, Xue Y, Liu D (2016). Neougonin A Inhibits Lipopolysaccharide-Induced Inflammatory Responses via Downregulation of the NF-kB Signaling Pathway in RAW 264.7 Macrophages. Inflammation.

[22] Wu Y, Li W, Zhou C, Lu F, Gao T, Liu Y, Cao J, Zhang Y, Zhang Y, Zhou C (2012). Ketamine inhibits lipopolysaccharide-induced astrocytes activation by suppressing TLR4/NF-kB pathway. Cell Physiol Biochem.

[23] Barham W, Chen L, Tikhomirov O, Onishko H, Gleaves L, Stricker TP, Blackwell TS, Yull FE (2015). Aberrant activation of NF-kappaB signaling in mammary epithelium leads to abnormal growth and ductal carcinoma *in situ*. BMC Cancer.

[24] Zhong W, Qian K, Xiong J, Ma K, Wang A, Zou Y (2016). Curcumin alleviates lipopolysaccharide induced sepsis and liver failure by suppression of oxidative stress-related inflammation via PI3K/AKT and NF-kappaB related signaling. Biomed Pharmacother.

[25] Spiga R, Marini MA, Mancuso E, Di FC, Fuoco A, Perticone F, Andreozzi F, Mannino GC, Sesti G (2017). Uric Acid Is Associated With Inflammatory Biomarkers and Induces Inflammation Via Activating the NF-kappaB Signaling Pathway in HepG2 Cells. Arterioscler Thromb Vasc Biol.

[26] Mateescu B, Batista L, Cardon M, Gruosso T, de FY, Mariani O, Nicolas A, Meyniel JP, Cottu P, Sastre-Garau X, Mechta-Grigoriou F (2011). miR-141 and miR-200a act on ovarian tumorigenesis by controlling oxidative stress response. Nat Med.

[27] Hinz M, Loser P, Mathas S, Krappmann D, Dorken B, Scheidereit C (2001). Constitutive NF-kappaB maintains high expression of a characteristic gene network, including CD40, CD86, and a set of antiapoptotic genes in Hodgkin/Reed-Sternberg cells. Blood.

[28] Guttridge DC, Albanese C, Reuther JY, Pestell RG, Baldwin AS (1999). NF-kappaB controls cell growth and differentiation through transcriptional regulation of cyclin D1. Mol Cell Biol.

[29] Yu Q, Geng Y, Sicinski P (2001). Specific protection against breast cancers by cyclin D1 ablation. Nature.

[30] Liu Z, Zhu G, Getzenberg RH, Veltri RW (2015). The Upregulation of PI3K/Akt and MAP Kinase Pathways is Associated with Resistance of Microtubule-Targeting Drugs in Prostate Cancer. J Cell Biochem.

[31] West KA, Castillo SS, Dennis PA (2002). Activation of the PI3K/Akt pathway and chemotherapeutic resistance. Drug Resist Updat.

[32] Zha L, Chen J, Sun S, Mao L, Chu X, Deng H, Cai J, Li X, Liu Z, Cao W (2014). Soyasaponins can blunt inflammation by inhibiting the reactive oxygen species-mediated activation of PI3K/Akt/NF-kB pathway. PLoS One.

[33] Jayasooriya RG, Lee KT, Lee HJ, Choi YH, Jeong JW, Kim GY (2014). Anti-inflammatory effects of beta-hydroxyisovalerylshikonin in BV2 microglia are mediated through suppression of the PI3K/Akt/NF-kB pathway and activation of the Nrf2/HO-1 pathway. Food Chem Toxicol.

[34] Rocha CR, Kajitani GS, Quinet A, Fortunato RS, Menck CF (2016). NRF2 and glutathione are key resistance mediators to temozolomide in glioma and melanoma cells. Oncotarget.

[35] Chen J, Solomides C, Simpkins F, Simpkins H (2017). The role of Nrf2 and ATF2 in resistance to platinum-based chemotherapy. Cancer Chemother Pharmacol.

[36] Bao L, Wu J, Dodson M, Rojo de la Vega EM, Ning Y, Zhang Z, Yao M, Zhang DD, Xu C, Yi X (2017). ABCF2, an Nrf2 target gene, contributes to cisplatin resistance in ovarian cancer cells. Mol Carcinog.

[37] Lu BC, Li J, Yu WF, Zhang GZ, Wang HM, Ma HM (2016). Elevated expression of Nrf2 mediates multidrug resistance in CD133+ head and neck squamous cell carcinoma stem cells. Oncol Lett.

[38] Ryoo IG, Kim G, Choi BH, Lee SH, Kwak MK (2016). Involvement of NRF2 Signaling in Doxorubicin Resistance of Cancer Stem Cell-Enriched Colonospheres. Biomol Ther Seoul.

[39] Xu Y, Luo Y, Wang ZY, Li X, Zheng P, Zhang TC (2017). MRTF-A can activate Nrf2 to increase the resistance to doxorubicin. Oncotarget.

[40] Zhong Y, Zhang F, Sun Z, Zhou W, Li ZY, You QD, Guo QL, Hu R (2013). Drug resistance associates with activation of Nrf2 in MCF-7/DOX cells, and wogonin reverses it by down-regulating Nrf2-mediated cellular defense response. Mol Carcinog.

[41] Khatri R, Shah P, Guha R, Rassool FV, Tomkinson AE, Brodie A, Jaiswal AK (2015). Aromatase Inhibitor-Mediated Downregulation of INrf2 (Keap1) Leads to Increased Nrf2 and Resistance in Breast Cancer. Mol Cancer Ther.

[42] Tsang WP, Kwok TT (2008). Let-7a microRNA suppresses therapeutics-induced cancer cell death by targeting caspase-3. Apoptosis.

[43] Piskounova E, Viswanathan SR, Janas M, LaPierre RJ, Daley GQ, Sliz P, Gregory RI (2008). Determinants of microRNA processing inhibition by the developmentally regulated RNA-binding protein Lin28. J Biol Chem.

[44] Viswanathan SR, Daley GQ, Gregory RI (2008). Selective blockade of microRNA processing by Lin28. Science.

