# Supplementary material for: OTUD7B upregulation predicts a poor response to paclitaxel in patients with triple-negative breast cancer
Source: Oncotarget. 2017 Dec 9;9(1):553–65. doi: 10.18632/oncotarget.23074 (PMC5787489; doi:10.18632/oncotarget.23074)
Supplement: Supplementary file 1 [file oncotarget-09-553-s001.pdf]

OTUD7B upregulation predicts a poor response to paclitaxel in patients with triple-negative breast cancer

SUPPLEMENTARY MATERIALS

Supplementary Table 1: List of consensus genes with 1.5-fold changes in MDA-MB436 and HCC38 cells following treatment with paclitaxel. See Supplementary_Table_1

Supplementary Table 2: List of consensus upstream regulators that are computationally predicted to be activated or inhibited after paclitaxel treatment in MDA-MB436 and HCC38 cells

MB436_Upstream Regulators	Activation z-score	*p*-value of overlap	HCC38_Upstream Regulators	Activation z-score	*p*-value of overlap
CSF2	2.186	7.71E-08	CSF2	3.725	1.19E-09
let-7	2.135	4.45E-09	let-7	–2.618	4.03E-06
lipopolysaccharide	–2.311	7.38E-04	lipopolysaccharide	2.537	6.21E-07
LY294002	2.052	1.57E-07	LY294002	–2.816	5.89E-07
MED1	2.359	1.78E-03	MED1	2.554	1.24E-04
PTGER2	2.714	3.98E-05	PTGER2	5.496	1.84E-21
S100A6	2.449	7.87E-03	S100A6	2.121	3.20E-04
SHH	–2.383	1.05E-04	SHH	–2.072	6.29E-02
STAT3	2.375	9.91E-08	STAT3	2.747	7.32E-08
TICAM1	–3.105	1.30E-05	TICAM1	2.191	4.47E-02
tretinoin	–2.444	7.06E-08	tretinoin	–2.255	2.42E-05

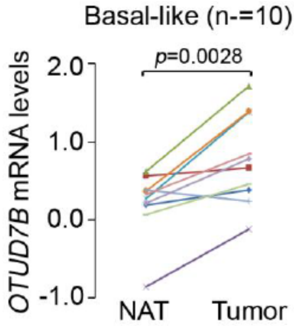

Supplementary Figure 1: *OTUD7B* expression is upregulated in primary tumors compared to normal adjacent tissues derived from patients with basal-like breast cancer. *OTUD7B* expression in paired normal adjacent tissues (NAT) and primary tumors derived from patients with BRCA was analyzed using TCGA database. The statistical difference was evaluated using paired *t*-tests.

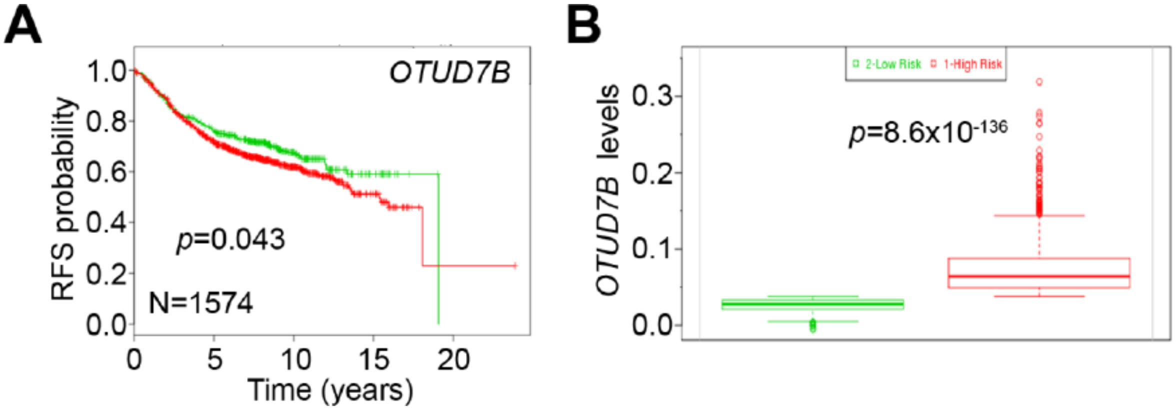

Supplementary Figure 2: *OTUD7B* upregulation predicts a poor recurrence-free survival probability in breast cancer patients. (**A**) Kaplan-Meier analysis of recurrence-free survival (RFS) probability according to *OTUD7B* expression in breast cancer patients, performed using the SurvExpress database. (**B**) Boxplot of *OTUD7B* mRNA levels in low (green) and high (red)-risk cohorts in A.

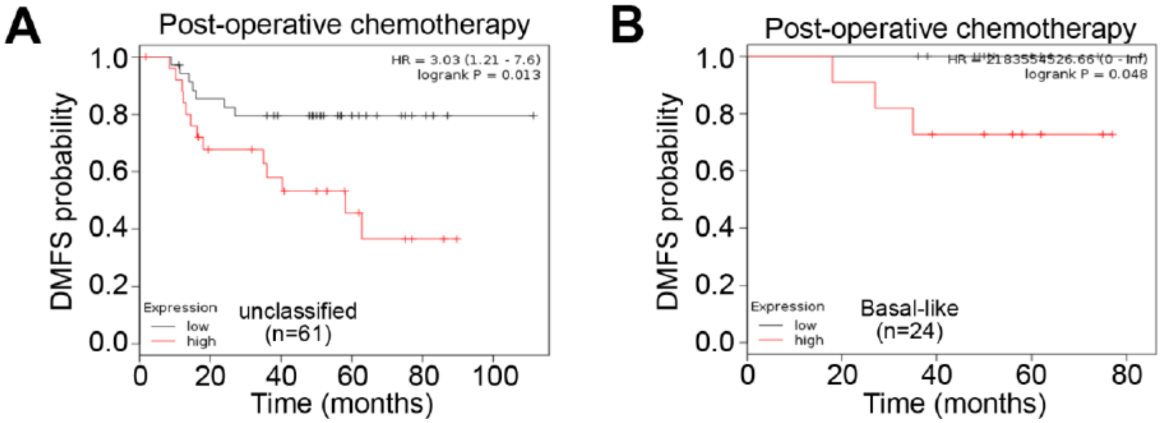

Supplementary Figure 3: *OTUD7B* upregulation increases the risk for cancer metastasis *n* breast cancer patients receiving post-operative paclitaxel chemotherapy. (**A**, **B**) K-M analysis of distant metastasis-free survival (DMFS) probability according to *OTUD7B* expression in patients who receive post-operative paclitaxel-based chemotherapy with unclassified (A) or basal-like (B) breast cancer. In (A, B) Kaplan-Meier analyses were performed using the K-M Plotter database and HR denotes hazard ratio.

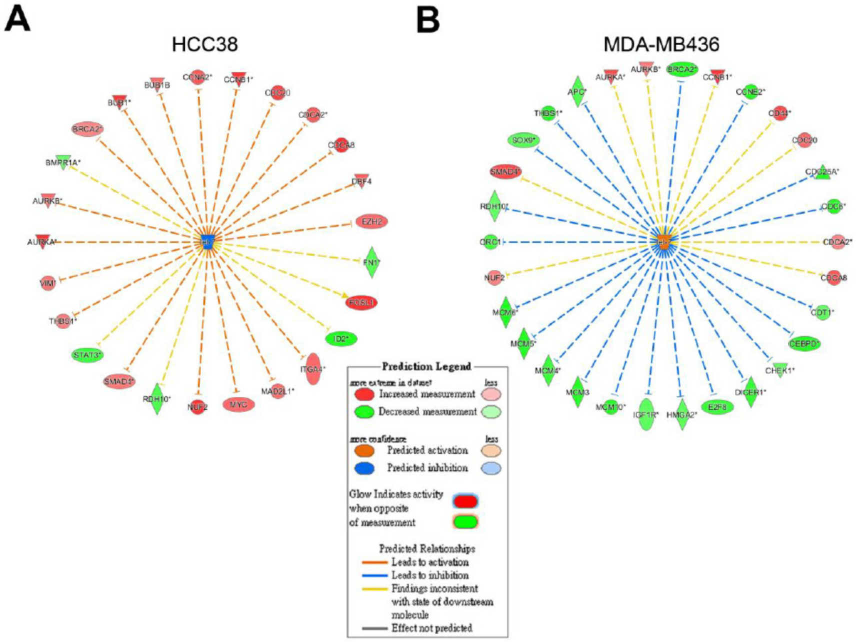

Supplementary Figure 4: Computational simulation of Let-7 targeting genes in paclitaxel-treated HCC38 and MDA-MB436 cells. (**A**, **B**) Computational simulation of Let-7 microRNA-targeting genes in HCC38 (A) and MDA-MB436 (B) cells following treatment with paclitaxel at 10 × IC_{50} for 24 hours.
