# Supplementary material for: OTUD7B upregulation predicts a poor response to paclitaxel in patients with triple-negative breast cancer
Source: Oncotarget. 2017 Dec 9;9(1):553–65. doi: 10.18632/oncotarget.23074 (PMC5787489; doi:10.18632/oncotarget.23074)
Supplement: Supplementary file 2 [file oncotarget-09-553-s002.docx]

**Supplementary Table 1: List of consensus genes with 1.5-fold changes in MDA-MB436 and HCC38 cells following treatment with paclitaxel**

| Probe ID | MB436 | HCC38 | Gene  Symbol | Probe ID | | MB436 | HCC38 | Gene Symbol |
| --- | --- | --- | --- | --- | --- | --- | --- | --- |
| 241925_x_at | -1.13852 | -0.8215 | SLC16A7 |  | 227249_at | 0.83158 | 0.818991 | NDE1 |
| 1555878_at | -0.99178 | -0.71606 | RPS24 |  | 233248_at | 0.83446 | 0.797113 |  |
| 225725_at | -0.92495 | -0.71691 | ZMAT3 |  | 215645_at | 0.838062 | 0.809586 | FLCN |
| 211325_x_at | -0.90261 | 0.71897 | DSTNP2 |  | 207286_at | 0.844755 | 0.992989 | CEP135 |
| 204619_s_at | -0.79859 | 0.638058 | VCAN |  | 228729_at | 0.844757 | 1.248961 | CCNB1 |
| 218807_at | -0.74588 | -0.93657 | VAV3 |  | 1559583_at | 0.845336 | 0.727667 | CD276 |
| 242940_x_at | -0.73726 | -0.63074 | DLX6 |  | 242612_at | 0.846835 | 0.925658 |  |
| 212192_at | -0.71157 | -0.70497 | KCTD12 |  | 205642_at | 0.855756 | 0.696891 | CNTRL |
| 202658_at | -0.70545 | -0.64251 | PEX11B |  | 238589_s_at | 0.857795 | 0.699528 |  |
| 224917_at | -0.6976 | -0.61684 | MIR21 |  | 201896_s_at | 0.859762 | 1.17792 | PSRC1 |
| 231285_at | -0.68769 | -0.70641 |  |  | 215013_s_at | 0.861753 | 0.671916 | USP34 |
| 230097_at | -0.67601 | -0.8341 | GART |  | 219544_at | 0.865152 | 1.079199 | BORA |
| 209348_s_at | -0.67298 | -0.99485 | MAF |  | 227420_at | 0.871259 | 1.158717 | TNFAIP8L1 |
| 223794_at | -0.65234 | -0.64399 | ARMC4 |  | 228252_at | 0.876299 | 1.15289 | PIF1 |
| 227221_at | -0.64866 | -0.6119 | ZMAT3 |  | 230998_at | 0.898903 | 0.773315 | CBX3 |
| 222787_s_at | -0.64032 | -0.67382 | TMEM106B | 236907_at | | 0.902492 | 0.789751 |  |
| 212454_x_at | -0.63549 | -0.67211 | HNRNPDL | 1560297_at | | 0.906966 | 0.70398 |  |
| 222108_at | -0.62474 | -0.66094 | AMIGO2 |  | 1553349_at | 0.919435 | 0.840885 | ARID2 |
| 207057_at | -0.62308 | -0.79659 | SLC16A7 |  | 243589_at | 0.939388 | 0.609509 | KANSL1 |
| 202094_at | 0.580624 | 1.205199 | BIRC5 |  | 1558783_at | 0.949837 | 0.821719 |  |
| 238840_at | 0.585888 | 1.046745 | LRRFIP1 |  | 230462_at | 0.949987 | 0.716789 | NUMB |
| 231292_at | 0.597426 | 1.003161 | EID3 |  | 219906_at | 0.950983 | 0.583897 | EBLN2 |
| 242726_at | 0.598982 | 0.907699 |  |  | 236168_at | 0.952221 | 0.791782 |  |
| 238994_at | 0.602818 | -0.58697 | OTUD7B |  | 233271_at | 0.953325 | 0.899017 |  |
| 221258_s_at | 0.603759 | 0.891615 | KIF18A |  | 238908_at | 0.955145 | 0.705455 |  |
| 204492_at | 0.616153 | 0.906507 | ARHGAP11A | 230077_at | | 0.957631 | 0.733572 | LOC220729 |
| 202227_s_at | 0.617962 | 0.657074 | BRD8 |  | 226840_at | 0.959692 | 0.629364 | H2AFY |
| 222625_s_at | 0.621308 | 1.185074 | NDE1 |  | 1559490_at | 0.966027 | 0.793822 | LRCH3 |
| 220221_at | 0.621506 | 0.65558 | VPS13D |  | 239243_at | 0.980352 | 0.658303 | ZNF638 |
| 243490_at | 0.622244 | 0.613583 |  |  | 230651_at | 0.990664 | 0.610241 |  |
| 209709_s_at | 0.622668 | 1.210764 | HMMR |  | 240146_at | 1.008641 | 0.87036 |  |
| 224783_at | 0.623041 | 0.972355 | UBALD2 |  | 214982_at | 1.025289 | 0.645224 | LOC101929 |
| 232344_at | 0.62726 | 0.698385 |  |  | 216697_at | 1.033916 | 1.180672 | TRIO |
| 232879_at | 0.628246 | 0.802205 | CRTC3 |  | 207711_at | 1.034904 | 1.50749 | SOGA1 |
| 221520_s_at | 0.62872 | 1.138309 | CDCA8 |  | 1559993_at | 1.035899 | 0.75135 | SFXN3 |
| 208079_s_at | 0.629637 | 1.112639 | AURKA |  | 207331_at | 1.039512 | 1.083421 | CENPF |
| 232958_at | 0.639913 | 0.648972 |  |  | 244427_at | 1.050581 | 0.664827 | KIF23 |
| 243050_at | 0.640431 | 0.594935 |  |  | 1558732_at | 1.057479 | 0.862373 | MAP4K4 |
| 235425_at | 0.643517 | 0.658041 | SGOL2 |  | 231552_at | 1.061807 | 0.812686 |  |
| 233869_x_at | 0.646513 | 0.680888 |  |  | 243012_at | 1.066841 | 0.72665 |  |
| 235709_at | 0.652724 | 0.794369 | GAS2L3 |  | 1554501_at | 1.075687 | 0.621642 | TSC22D4 |
| 238797_at | 0.655609 | 0.584562 | TRIM11 |  | 217659_at | 1.082077 | 0.720392 |  |
| 221922_at | 0.660853 | 0.797297 | GPSM2 |  | 238951_at | 1.087662 | 1.328863 |  |
| 238678_at | 0.669405 | 0.762053 | LINC01000 | 228582_x_at | | 1.089438 | 1.069108 | MALAT1 |
| 231985_at | 0.670793 | 0.968754 | MICAL3 |  | 214805_at | 1.136024 | 0.733866 | EIF4A1 |
| 215623_x_at | 0.678588 | 0.918982 | SMC4 |  | 242550_at | 1.1361 | 0.824752 | EIF3B |
| 229485_x_at | 0.678735 | 0.839029 | SHISA3 |  | 223494_at | 1.139359 | 0.698641 | MGEA5 |
| 218726_at | 0.679125 | 0.874335 | HJURP |  | 242265_at | 1.144881 | 1.103835 | BRD8 |
| 1565703_at | 0.681813 | 0.584404 | SMAD4 |  | 243751_at | 1.162453 | 1.704419 | CHD2 |
| 1559731_x_at | 0.683645 | 0.642905 | MACROD1 | 229514_at | | 1.164987 | 1.621474 | GPATCH2L |
| 210821_x_at | 0.684407 | 1.091816 | CENPA |  | 242431_at | 1.174934 | 1.005211 |  |
| 1557581_x_at | 0.685368 | 0.820812 |  |  | 215123_at | 1.185637 | 1.082877 | LOC101929 |
| 204092_s_at | 0.686637 | 1.079576 | AURKA |  | 1552729_at | 1.201111 | 0.875789 | SNHG7 |
| 215942_s_at | 0.700825 | 0.973674 | GTSE1 |  | 1557580_at | 1.203119 | 0.781083 |  |
| 204315_s_at | 0.701195 | 1.114351 | GTSE1 |  | 238156_at | 1.232332 | 1.415982 |  |
| 239071_at | 0.703424 | 0.585461 | RBBP4 |  | 233303_at | 1.235442 | 1.149851 |  |
| 239629_at | 0.717668 | 0.675156 |  |  | 242261_at | 1.236055 | 0.627001 | IREB2 |
| 204962_s_at | 0.719761 | 1.067908 | CENPA |  | 215190_at | 1.237845 | 0.833752 | EIF3M |
| 242922_at | 0.725803 | 0.696377 | NOMO3 |  | 235959_at | 1.255676 | 0.89796 |  |
| 220295_x_at | 0.728436 | 0.979666 | DEPDC1 |  | 242751_at | 1.257591 | 1.828358 |  |
| 215224_at | 0.729633 | 0.968997 | SNORA21 |  | 243046_at | 1.259673 | 1.525747 |  |
| 243765_at | 0.729939 | 0.703047 |  |  | 240221_at | 1.274487 | 0.967548 | CSNK1A1 |
| 1556007_s_at | 0.732486 | 1.046665 | CSNK1A1 |  | 1563075_s_at | 1.330384 | 1.151754 |  |
| 240247_at | 0.737819 | 0.709073 |  |  | 239228_at | 1.347395 | 0.865889 |  |
| 1561130_at | 0.737994 | 1.130983 | HECTD4 |  | 224559_at | 1.380897 | 2.118033 | MALAT1 |
| 242298_x_at | 0.751731 | 0.79054 |  |  | 1569519_at | 1.401838 | 0.75155 | LOC102724 |
| 215558_at | 0.75226 | 1.596961 | UBR2 |  | 1557238_s_at | 1.414674 | 1.021167 |  |
| 1558750_a_at | 0.75234 | 1.097741 | ARHGAP11B | 239937_at | | 1.417656 | 1.751382 | ZNF207 |
| 208015_at | 0.754091 | 0.600523 | SMAD1 |  | 215066_at | 1.417779 | 1.108984 | PTPRF |
| 243338_at | 0.767258 | 0.614817 | CSNK1A1 |  | 1559529_at | 1.432449 | 0.63942 | PTK2 |
| 216682_s_at | 0.771486 | 1.160269 | SUPT20H |  | 223679_at | 1.452806 | 1.712765 | CTNNB1 |
| 210469_at | 0.776352 | 0.618396 | DLG5 |  | 239121_at | 1.50926 | 1.229851 |  |
| 204649_at | 0.777203 | 1.211789 | TROAP |  | 242111_at | 1.556906 | 0.92894 | METTL3 |
| 232362_at | 0.779369 | 0.608042 | CCDC18 |  | 242673_at | 1.566954 | 0.602676 |  |
| 204317_at | 0.783053 | 0.612596 | GTSE1 |  | 241242_at | 1.677216 | 0.92667 |  |
| 215374_at | 0.796276 | 0.660954 | PAPOLA |  | 216109_at | 1.815029 | 1.32501 | MED13L |
| 207728_at | 0.808239 | 0.912131 | ATF7IP |  | 241387_at | 1.924125 | 1.109965 |  |
| 1563076_x_at | 0.812863 | 0.792235 |  |  | 215175_at | 2.093647 | 2.07459 | PCNX |
| 244185_at | 0.820088 | 0.987531 |  |  | 244766_at | 2.110755 | 1.992106 | BOLA2 |
